# Psychological and Clinical Factors Mediate Post‐COVID‐19 Irritable Bowel Syndrome

**DOI:** 10.1111/nmo.70079

**Published:** 2025-05-15

**Authors:** Keren Hod, Giovanni Marasco, Luigi Colecchia, Cesare Cremon, Maria Raffaella Barbaro, Giulia Cacciari, Francesca Falangone, Anna Kagramanova, Dmitry Bordin, Vasile Drug, Egidia Miftode, Pietro Fusaroli, Salem Youssef Mohamed, Chiara Ricci, Massimo Bellini, M. Masudur Rahman, Luigi Melcarne, Javier Santos, Beatriz Lobo, Serhat Bor, Suna Yapali, Deniz Akyol, Ferdane Pirincci Sapmaz, Yonca Yilmaz Urun, Tugce Eskazan, Altay Celebi, Huseyin Kacmaz, Berat Ebik, Hatice Cilem Binicier, Mehmet Sait Bugdayci, Munkhtsetseg Banzragch Yağcı, Husnu Pullukcu, Berrin Yalınbas Kaya, Ali Tureyen, İbrahim Hatemi, Elif Sitre Koc, Goktug Sirin, Ali Riza Calıskan, Goksel Bengi, Esra Ergun Alıs, Snezana Lukic, Meri Trajkovska, Dan Dumitrascu, Antonello Pietrangelo, Elena Corradini, Magnus Simren, Jessica Sjolund, Navkiran Tornkvist, Uday C. Ghoshal, Olga Kolokolnikova, Antonio Colecchia, Jordi Serra, Giovanni Maconi, Roberto De Giorgio, Silvio Danese, Piero Portincasa, Antonio Di Sabatino, Marcello Maggio, Elena Philippou, Yeong Yeh Lee, Daniele Salvi, Alessandro Venturi, Claudio Borghi, Marco Zoli, Paolo Gionchetti, Pierluigi Viale, Vincenzo Stanghellini, Giovanni Barbara, GI‐COVID19 study group

**Affiliations:** ^1^ Department of Nutritional Sciences School of Health Sciences, Ariel University Ariel Israel; ^2^ Assuta Medical Centers Tel Aviv Israel; ^3^ Department of Medical and Surgical Sciences University of Bologna Italy; ^4^ IRCCS Azienda Ospedaliero‐Universitaria di Bologna Bologna Italy; ^5^ Medical‐Surgical Department of Clinical Sciences and Translational Medicine University Sapienza Rome Italy; ^6^ A. S. Loginov Moscow Clinical Scientific Center Moscow Russia; ^7^ Research Institute of Health Organization and Medical Management Moscow Russia; ^8^ Tver State Medical University Tver Russia; ^9^ Russian University of Medicine Moscow Russia; ^10^ Department of Gastroenterology ‘Grigore T. Popa’ University of Medicine and Pharmacy Iasi Romania; ^11^ Department of Infectious Diseases ‘Grigore T. Popa’ University of Medicine and Pharmacy Iasi Romania; ^12^ Gastroenterology Unit Imola Hospital Imola Italy; ^13^ Gastroenterology and Hepatology Unit, Internal Medicine Department Faculty of Medicine, Zagazig University Egypt; ^14^ Department of Experimental and Clinical Sciences University of Brescia, Spedali Civili di Brescia Brescia Italy; ^15^ Gastroenterology Unit University of Pisa Pisa Italy; ^16^ Sheikh Russel National Gastroliver Institute and Hospital Dhaka Bangladesh; ^17^ Hospital Universitari Parc Taulí, Sabadell—CIBEREHD Centro de Investigación Biomédica en Red Spain; ^18^ Gastroenterology Department Vall d'Hebron Hospital Universitari, Vall d'Hebron Hospital Campus Barcelona Spain; ^19^ Digestive Physiology and Physiopathology Research Group Vall d'Hebron Research Institute (VHIR) Barcelona Spain; ^20^ Centro de Investigación Biomédica en Red, Enfermedades Hepáticas y Digestivas (CIBERhed), Instituto de Salud Carlos III Madrid Spain; ^21^ Ege University Division of Gastroenterology Izmir Turkey; ^22^ Acibadem University, Altunizade Acibadem Hospital Division of Gastroenterology Istanbul Turkey; ^23^ Ege University Department of Infectious Diseases Izmir Turkey; ^24^ University of Health Sciences, Keciören Education and Research Hospital Division of Gastroenterology Keciören Turkey; ^25^ Eskisehir City Hospital Division of Gastroenterology Eskisehir Turkey; ^26^ Istanbul University‐Cerrahpasa, Cerrahpasa Faculty of Medicine Division of Gastroenterology Turkey; ^27^ Kocaeli University Division of Gastroenterology Kocaeli Turkey; ^28^ Adiyaman Education and Research Hospital Division of Gastroenterology Adiyaman Turkey; ^29^ University of Health Sciences, Diyabakır Gazi Yasargil Education and Research Hospital Division of Gastroenterology Diyarbakır Turkey; ^30^ Dokuz Eylül University Division of Gastroenterology Izmir Turkey; ^31^ İstanbul Aydın University Florya Liv Hospital Division of Gastroenterology Istanbul Turkey; ^32^ Darıca Farabi Education and Research Hospital Division of Gastroenterology Kocaeli Turkey; ^33^ İstanbul Aydın University Florya Liv Hospital Department of Infectious Diseases Istanbul Turkey; ^34^ Clinic for Gastroenterohepatology University Clinical Centre of Serbia Belgrade Serbia; ^35^ Clinic of Gastroenterohepatology Skopje North Macedonia; ^36^ Iuliu Hatieganu University of Medicine and Pharmacy Cluj‐Napoca Romania; ^37^ Internal Medicine Unit Modena University Hospital, University of Modena and Reggio Emilia Modena Italy; ^38^ Sahlgrenska University Hospital Gothenburg Sweden; ^39^ Institute of Gastrosciences and Liver Transplantation Apollo Multispeciality Hospitals Kolkata India; ^40^ Medsi Clinical Hospital Russia; ^41^ Gastroenterology Unit Verona University Hospital Verona Italy; ^42^ CIBERehd, University Hospital Germans Trias i Pujol Barcelona Spain; ^43^ Gastroenterology Unit Department of Biomedical and Clinical Sciences, L.Sacco University Hospital, University of Milan Milan Italy; ^44^ Department of Translational Medicine University of Ferrara Ferrara Italy; ^45^ Gastroenterology and Endoscopy IRCCS Ospedale San Raffaele and University Vita‐Salute San Raffaele Milano Italy; ^46^ Department of Precision and Regenerative Medicine and Ionian Area (DiMePre‐J). University of Bari “Aldo Moro”, Bari Division of Internal Medicine “A. Murri” Italy; ^47^ First Department of Internal Medicine Fondazione IRCCS Policlinico San Matteo, University of Pavia Pavia Italy; ^48^ Geriatric Clinic Unit, Medical Geriatric Rehabilitative Department University Hospital of Parma Parma Italy; ^49^ Department of Life Sciences School of Life and Health Sciences, University of Nicosia Cyprus; ^50^ School of Medical Sciences, Universiti Sains Malaysia Kota Bharu Malaysia; ^51^ O.M. Filatov Municipal Clinical Hospital no 15 Moscow Russia; ^52^ Clinic for Infectious and Tropical Diseases University Clinical Centre of Serbia Belgrade Serbia; ^53^ Meir Hospital Kfar Saba Israel; ^54^ Azienda Ospedaliero‐Universitaria di Bologna, Istituto di Ematologia “Seràgnoli”, Dipartimento di Medicina Specialistica, Diagnostica e Sperimentale Università di Bologna Italy; ^55^ Gastroenterology Department Hospital del Mar Barcelona Spain; ^56^ Department of Experimental and Clinical Biomedical Science AOU Careggi, University of Florence Italy; ^57^ Transilvania University, Faculty of Medicine Brasov Romania; ^58^ Clinic of Infectology Skopje North Macedonia; ^59^ Humanitas Hospital Milan Italy

**Keywords:** COVID‐19, depression, dyspnea, gastrointestinal symptoms, irritable bowel syndrome, mediation analysis

## Abstract

**Background:**

Exposure to COVID‐19 has been shown previously to be associated with a higher risk for irritable bowel syndrome (IBS). This study aimed to better explain this relationship using mediation analysis.

**Methods:**

This post hoc analysis of a multicenter cohort study includes 623 patients with and without COVID‐19 infection. All participants completed the ROME IV criteria, gastrointestinal symptom rating scale (GSRS), and hospital anxiety and depression scale (HADS) over 1 year. Mediation analysis utilized the PROCESS macro and Baron and Kenny's method for parametric and nonparametric mediating variables, respectively.

**Key Results:**

The impact of COVID‐19 on the development of post‐COVID‐19 IBS is completely mediated by dyspnea at baseline (adjusted OR = 3.561, *p* = 0.012), severity of acid regurgitation at 1 month [indirect effect, log‐odds metric = 0.090, 95% CI (0.006–0.180)], hunger pains at 1 [indirect effect, log‐odds metric = 0.094, 95% CI (0.024–0.178)], and 6 months [indirect effect, log‐odds metric = 0.074, 95% CI (0.003–0.150)], depression at 6 [indirect effect, log‐odds metric = 0.106, 95% CI (0.009–0.225)] and 12 months [indirect effect, log‐odds metric = 0.146, 95% CI (0.016–0.311)] as well as borborygmus [indirect effect, log‐odds metric = 0.095, 95% CI (0.009–0.203)], abdominal distention [indirect effect, log‐odds metric = 0.162, 95% CI (0.047–0.303)], and increased flatus [indirect effect, log‐odds metric = 0.110, 95% CI (0.005–0.234)] at 12 months.

**Conclusions and Inferences:**

Our findings provide evidence for psychological and clinical mediators between COVID‐19 and post‐COVID‐19 IBS, which may be promising targets for interventions tailored for treating or preventing depression. The presence of specific GI symptoms at COVID‐19 onset and their persistence should increase awareness of a potential new onset of IBS diagnosis.

## Graphical abstract

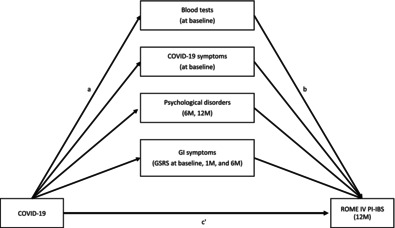

**Summary**

- COVID‐19 exposure has been linked to a higher risk of developing irritable bowel syndrome (IBS), but the exact reasons behind this are not fully understood.
- The development of post‐COVID‐19 IBS is influenced by breathing difficulties (dyspnea), depression, and specific gastrointestinal symptoms like acid reflux and hunger pains.
- Addressing depression and persistent gastrointestinal symptoms after COVID‐19 could help prevent or manage IBS. These findings highlight the importance of early detection and targeted interventions.

## 1 Introduction

The COVID‐19 pandemic has profoundly impacted global health, with over 775 million cases and over 7 million deaths as of December 2024 [1].

Severe acute respiratory syndrome coronavirus 2 (SARS‐CoV‐2) infection typically presents respiratory symptoms including cough, fever, and shortness of breath. Moreover, approximately 10% of SARS‐CoV‐2 infections result in long COVID, a frequently debilitating illness, subclassified as ongoing symptomatic COVID‐19 (4–12 weeks of symptoms) and post‐COVID‐19 syndrome (PCS, 12 weeks or more) [2], affecting an estimated > 65 million people worldwide [3]. Over 200 symptoms impacting multiple organs have been identified, including the typically reported respiratory symptoms (e.g., dyspnea, cough), as well as arthralgia, fatigue, chest pain, cognitive abnormalities, and reduced quality of life [4]. COVID‐19 also affects gastrointestinal (GI) health, with about 20% of patients reporting symptoms such as abdominal pain, anorexia, diarrhea, and vomiting [5].

We previously explored the link between COVID‐19 and irritable bowel syndrome (IBS), finding that IBS and other chronic GI symptoms were more likely to develop in patients recovering from acute COVID‐19 infection. According to Rome IV criteria, the prevalence of IBS is higher in COVID‐19 survivors than in the general population (3.2% vs. 0.5%) [6]. This finding was later supported by a meta‐analysis of 10 studies with 2763 COVID‐19 patients, showing an increased odds ratio (OR = 6.27, 95% CI: 0.88–44.76) for IBS development in COVID‐19 cases [7].

The mediating factors underlying the link between COVID‐19 and IBS are not fully elucidated. However, SARS‐CoV‐2 is known to infect the GI tract by binding to angiotensin‐converting enzyme 2 (ACE2) receptors in the gut, which triggers immune responses and inflammation that may contribute to IBS development [8]. Altered gut microbiota in COVID‐19 patients also disrupts the gut–brain pathway, potentially leading to IBS‐associated symptoms such as anxiety or depression [9].

Previous data identified predictive factors for IBS in COVID‐19 patients, including allergy history, proton pump inhibitor use, and dyspnea [6]. However, specific mediating factors remain unclear. To better explain this relationship, we performed a mediation analysis to assess how COVID‐19 may influence IBS development through potential mediating factors.

## 2 Materials and Methods

### 2.1 Study Population and Design

This post hoc analysis is based on a previous multicenter cohort study investigating long‐term post‐COVID‐19 GI symptoms and disorders of gut–brain interaction (DGBI) in 2183 hospitalized patients, including those with COVID‐19 and a control group without infection. Participants were prospectively recruited during the early pandemic period (from May to October 2020) at the time of admission (baseline) and followed up at 1, 6, and 12 months, across 36 centers in 14 countries: Italy, Bangladesh, Cyprus, Egypt, Israel, India, Macedonia, Malaysia, Romania, the Russian Federation, Serbia, Spain, Sweden, and Turkey [6, 10]. Inclusion criteria included ages 18–85, positive or negative for COVID‐19 with severe symptoms enough to require hospitalization, no preexisting GI symptoms at least 6 months before enrollment, and no history of other GI disorders or surgeries. The control group comprised patients hospitalized for non‐COVID‐19 reasons and enrolled within the study timeframe in internal medicine units of participating centers. All patients provided written informed consent.

### 2.2 Measurements and Evaluations

Demographic information, medical history, laboratory tests [i.e., blood count, international normalized ratio (INR), creatinine, aspartate amino transferase (AST), alanine amino transferase (ALT), gamma glutamyl transferase (GGT), ferritin, interleukin 6 (IL‐6) and C‐reactive protein (CRP)] and clinical data were collected from patients' medical files. COVID‐19 was diagnosed per WHO guidelines [11]. At baseline, patients were assessed for COVID‐19‐related symptoms. IBS diagnosis was determined using ROME IV at 6 and 12 months [12]. The gastrointestinal symptom rating scale (GSRS) was administered at four time points for the exclusion process (before admission) and for the evaluation of GI symptoms severity (at baseline, and at 1 and 6 months) [13]. Depression and anxiety were assessed using the hospital anxiety and depression scale (HADS) at 6 and 12 months [14].

### 2.3 Endpoints

**FIGURE 1:**
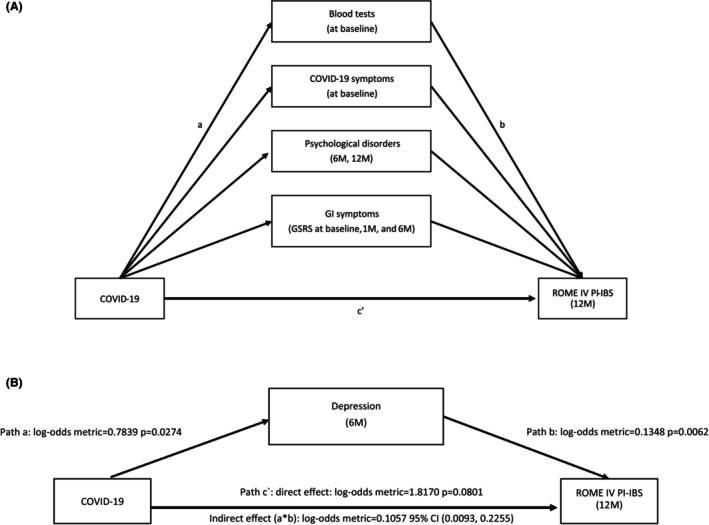
(A) Postulated mediational path model. The conceptual framework of the mediation model of the current study: Post‐COVID‐19 IBS results from the direct effect of COVID‐19 (Path c') as well as the mediating variables (Paths b's). At the same time, there is an impact of COVID‐19 on mediating variables (Path a's). When the indirect effect (a × b) is significant while Path c' effect is not, we claim complete mediation; when the indirect effect (a × b) is significant as well as Path c' effect, we claim partial mediation. (B) Example of depression as a mediating variable that completely mediates the development of postinfectious IBS. Post‐COVID‐19 IBS results from the direct effect of COVID‐19 (Path c') as well as depression (Path b). At the same time, there is an impact of COVID‐19 on depression (Path a). The indirect effect (a × b) is significant (95% CI 0.0093–0.2255) while Path c' effect is not (*p* = 0.0740), and therefore, we claim a complete mediation.

The primary endpoint was to assess whether the relationship between COVID‐19 and post‐COVID‐19 IBS is mediated via psychological (i.e., anxiety, depression) or clinical factors (i.e., baseline laboratory test results, COVID‐19‐related symptoms, and GI symptoms at three time points) (Figure 1A,B). Only GI symptoms not included in the IBS definition, such as hunger pains, acid regurgitation, nausea, heartburn, borborygmus, eructation, increased flatulence, and distention, were considered as mediating variables, while IBS‐defining symptoms were excluded. For mediation models involving psychological factors (depression and anxiety), participants who reported preexisting psychological disorders or psychiatric illnesses were excluded from the analysis to increase the likelihood that the model reflects causal relationships.

### 2.4 Statistical Analysis

Descriptive analyses for demographic, patients’ history, and psychological disorders are reported as median (interquartile range, IQR) for continuous nonnormally distributed variables, and as frequency and percentages for categorical variables.

Chi square and Mann–Whitney tests compared demographic, psychological, and clinical characteristics between COVID‐19 and control groups.

A mediation analysis was performed to investigate the main hypothesis of the postulated mediational path model, by which the effect of COVID‐19 upon post‐COVID‐19 IBS operates, completely or in part, through several mediators (Figure 1A). For continuous mediators (i.e., psychological scores, GI symptoms severity, and baseline laboratory results), a simple mediation analysis was conducted using the PROCESS macro for SPSS with bootstrapping techniques with a 95% CI based on 5000 samples (version 4.2; Andrew F. Hayes 2022). Since post‐COVID‐19 IBS is a binary outcome, mediation effects are expressed in log‐odds, indicating the likelihood of developing post‐COVID‐19. For nonparametric mediators (i.e., presence of COVID‐19 related symptoms at baseline) we used the Baron and Kenny's method [15], involving three univariate regression models that tested the relationships for each path: a, b, and c', as presented in Figure 1A,B. In the final step, multivariate regression assessed COVID‐19 and mediators (i.e., COVID‐19 related symptoms) as predictors of post‐COVID‐19 IBS. Full mediation is considered if the COVID‐19‐IBS relationship (Path c') became nonsignificant when controlling for mediators, while partial mediation implied a reduced relationship. A total of 47 models were tested, consistently using COVID‐19 diagnosis at baseline as the predictor and post‐COVID‐19 IBS at 12 months as the outcome, with different mediators in each model (10 for laboratory tests, 9 for COVID‐19 symptoms, 4 for psychological scores at 6, 12 months, and 24 for GSRS at three time points). All analyses used two‐tailed significance (*p* < 0.05). The SPSS statistical package (Version 30, SSPS Inc., Chicago, IL) was used for all analyses.

## 3 Results

### 3.1 Study Population

**FIGURE 2:**
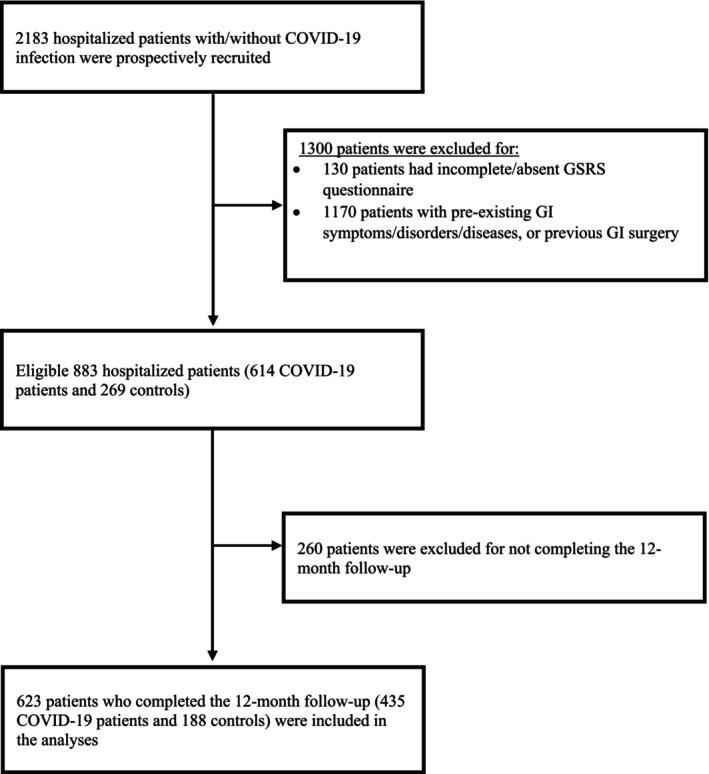
Flow chart of the selection of patients enrolled in the study. Controls were defined as patients without COVID‐19 infection. Abbreviations: GI, Gastrointestinal; GSRS, Gastrointestinal Symptoms Rating Scale.

Of the 2183 prospectively recruited hospitalized patients with or without (controls) COVID‐19 infection, 1300 patients were excluded, and 883 patients (614 COVID‐19 patients and 269 controls) were eligible for inclusion (Figure 2). Of 883 patients, 623 patients (435 COVID‐19 patients and 188 controls) who completed 12‐month follow‐up were analyzed (Figure 2). The demographic information and the anamnestic population characteristics are summarized in Table 1. There were no significant differences between the population groups except for smoking status (*p* < 0.001) and alcohol consumption (*p* = 0.032). Details on comorbidities and chronic drugs are reported in Table S1. Of the 623 patients, 23 (3.7%) reported preexisting psychological disorders or psychiatric diseases prior to enrollment. This included 10 patients with psychological disorders, 12 with psychiatric diseases, and one patient with both, with no significant differences observed between the groups (Table S1). These 23 patients were excluded solely from the mediation models involving psychological mediators to allow for a more robust examination of causality.

**TABLE 1:** Demographics, anamnestic characteristics, and psychological disorders of the study population.

Demographics and anamnestic characteristics	Total population (*n* = 623), *n* (%) or median (IQR)	Controls (*n* = 188), *n* (%) or median (IQR)	COVID‐19 patients (*n* = 435), *n* (%) or median (IQR)	*p*
Age (years)	51.0 (36.0–62.0)	49.0 (34.0–64.0)	52.0 (37.0–61.0)	0.981
Sex, male	369 (60.1)	114 (61.6)	255 (59.4)	0.654
BMI (kg/m^2^)	26.8 (24.0–30.9)	26.4 (23.7–30.9)	26.8 (24.1–30.9)	0.323
Smoker				< 0.001
No	396 (64.0)	88 (46.8)	308 (71.5)	
Current	95 (15.3)	56 (29.8)	39 (9.0)	
Former	128 (20.7)	44 (23.4)	84 (19.5)	
Alcohol consumption	114 (18.5)	44 (23.7)	70 (16.3)	0.032
Physical activity (at least 30 min three times/week)	202 (33.9)	64 (34.2)	138 (33.8)	0.926
Laboratory tests				
Lymphocyte count (10^3^/mm^3^)	1.4 (0.9–2.1)	1.7 (1.2–2.5)	1.3 (0.9–1.8)	< 0.001
Platelet count (10^3^/mm^3^)	216.0 (166.0–273.8)	254.5 (207.3–303.8)	200.5 (161.0–254.8)	< 0.001
INR	1.0 (0.9–1.1)	1.0 (0.9–1.2)	1.0 (0.9–1.1)	0.373
Creatinine (mg/dl)	0.8 (0.7–1.0)	0.9 (0.7–1.1)	0.8 (0.7–1.0)	0.022
AST (UI/L)	27.0 (19.0–42.0)	21.5 (15.0–34.2)	29.0 (21.0–43.5)	< 0.001
ALT (UI/L)	27.0 (17.0–43.0)	20.0 (14.0–39.2)	29.0 (19.0–44.0)	< 0.001
GGT (UI/L)	36.0 (21.0–70.0)	36.0 (19.0–81.5)	35.0 (22.0–63.0)	0.737
Ferritin (mg/l)	242.0 (98.0–514.0)	159.0 (60.9–414.5)	274.2 (105.5–538.3)	0.010
IL‐6 (pg/ml)	28.6 (11.9–71.7)	79.0 (18.4–348.9)	25.0 (11.6–59.3)	< 0.001
C‐reactive protein (mg/dl)	3.3 (0.8–9.9)	1.3 (0.4–6.9)	4.1 (1.0–11.2)	< 0.001
COVID‐19–related symptoms (at baseline)				
Fever	328 (52.6)	20 (10.6)	308 (70.8)	< 0.001
Fatigue	260 (41.7)	20 (10.6)	240 (55.2)	< 0.001
Cough	257 (41.3)	16 (8.5)	241 (55.4)	< 0.001
Myalgia	168 (27.0)	11 (5.9)	157 (36.1)	< 0.001
Dyspnea	157 (25.2)	21 (11.2)	136 (31.3)	< 0.001
Runny nose	35 (5.6)	2 (1.1)	33 (7.6)	< 0.001
Headaches	137 (22.0)	7 (3.7)	130 (29.9)	< 0.001
Anosmia	117 (18.8)	2 (1.1)	115 (26.4)	< 0.001
Dysgeusia	103 (16.5)	3 (1.6)	100 (23.0)	< 0.001
Psychological disorders at 6 and 12 months of follow‐up				
Depression score at 6 months	2.0 (0.0–5.0)	1.0 (0.0–4.0)	2.0 (0.0–6.0)	0.020
Depression score at 12 months	3.0 (0.0–5.0)	1.0 (0.0–3.0)	2.0 (0.0–5.0)	0.020
Anxiety score at 6 months	1.0 (0.0–4.0)	3.0 (0.0–5.0)	3.0 (0.0–5.0)	0.213
Anxiety score at 12 months	2.0 (0.0–5.0)	1.0 (0.0–1.0)	2.0 (0.0–5.0)	0.078

*Note:* Controls were defined as patients without COVID‐19 infection. All patients without preexisting GI symptoms, disorders or diseases or previous GI surgery. Anxiety and depression were evaluated by HADS (hospital anxiety and depression scale).

Abbreviations: ALT, alanine amino transferase; AST, aspartate amino transferase; BMI, body mass index; GGT, gamma glutamyl transferase; IL‐6, interleukin 6; INR, international normalized ratio; IQR, interquartile range; n, number.

### 3.2 Mediating Variables: Laboratory Tests at Baseline

The levels of lymphocytes, platelets, creatinine, and IL‐6 were significantly lower (*p* < 0.001, *p* < 0.001, *p* = 0.022, *p* < 0.001, respectively), and the levels of ALT, AST, ferritin, and CRP were significantly higher (*p* < 0.001, *p* < 0.001, *p* = 0.010, *p* < 0.001, respectively) among COVID‐19 patients compared to the control group (Table 1). The mediation analysis revealed that all blood tests had a nonsignificant indirect effect (a × b) (Table S2). Therefore, no laboratory test was identified as a mediating variable between COVID‐19 and post‐COVID‐19 IBS.

### 3.3 Mediating Variables: COVID‐19–Related Symptoms at Baseline

As expected, all COVID‐19–related symptoms were more common among the COVID‐19 patients as compared to the control group (all *p* < 0.001) (Table 1). Mediation analyses examined COVID‐19–related symptoms (i.e., fever, fatigue, cough, myalgia, dyspnea, runny nose, headache, anosmia, and dysgeusia) (Table S3). Among these, only fever, cough, and dyspnea were significantly associated with the relationship between COVID‐19 (Path a) and post‐COVID‐19 IBS (Path b) based on unstandardized regression coefficients. However, the relationship between COVID‐19 and post‐COVID‐19 IBS was eliminated entirely when dyspnea (mediating variable) was controlled (i.e., Path c' is nonsignificant). In contrast, when adjusting for fever or cough, the relationship between COVID‐19 and post‐COVID‐19 IBS (Path c') was eliminated entirely, as well as the relationship between fever or cough and post‐COVID‐19 IBS (b). Therefore, it can be concluded that the relationship between COVID‐19 and post‐COVID‐19 IBS was completely mediated by the presence of dyspnea (Table 2).

**TABLE 2:** Simple mediation analyses for the relationship between COVID‐19 and post‐COVID‐19 IBS (for nonparametric mediating variables), significant parameters.

Mediating variables (M)	First step: OR of COVID‐19 on M (a)	Second step: OR of M on post‐COVID‐19 IBS (b)	Third step: OR of COVID‐19 on post‐COVID‐19 IBS (c')	Fourth step: ORs of the adjusted model (b, c')	Interpretation
Dyspnea at baseline	3.617***	4.461**	7.141*	3.561*, 5.172	Complete mediation effect

*Note:* *p < 0.05. **p < 0.01. ***p < 0.001. All analyses conducted using the Baron and Kenny's method with bootstrapping techniques with 95% CI based on 5000 samples.

Abbreviations: IBS, irritable bowel syndrome; M, mediating variable; OR, odds‐ratio.

### 3.4 Mediating Variables: Psychological Disorders at 6 and 12 Months

As shown in Table 1, depression scores at both 6 and 12 months were significantly higher among COVID‐19 patients compared to controls (median score: 2.0 vs. 1.0, *p* = 0.020 for both time points), whereas anxiety scores did not differ between the groups. These findings remained consistent even after excluding 23 patients with preexisting psychological disorders or psychiatric diseases.

The results of the mediation analysis revealed a significant indirect effect (a × b, as depicted in Figure 1B) of depression at 6 and 12 months (log‐odds metric = 0.106, 95% CI 0.009–0.225; log‐odds metric = 0.146, 95% CI 0.016–0.311, respectively). In contrast, the direct effect of COVID‐19 on post‐COVID‐19 IBS (c') was not significant in the presence of depression at these two time points (log‐odds metric = 1.817, 95% CI −0.218–3.852; log‐odds metric = 1.749, 95% CI −0.292–3.791, respectively). Hence, it can be concluded that depression at 6 and 12 months was a complete mediating variable between COVID‐19 and post‐COVID‐19 IBS (Table 3, Figure 1B). Anxiety scores at both 6 and 12 months did not meet the mediation criteria and, thus, anxiety was not considered a mediating variable at any time point (Table S2).

**TABLE 3:** Simple mediation analyses for the relationship between COVID‐19 and post‐COVID‐19 IBS (for parametric mediating variables), significant parameters.

Mediating variables (M)	Effect of COVID‐19 on M (a)	Effect of M on post‐COVID‐19 IBS (b)	Direct effect (c')	Indirect effect (a × b)	Mediation effect	Percentage of mediation effect of the total effect
Psychological disorders						
Depression at 6 months	0.784*	0.135**	1.817	0.106^a^	Complete	5.51%
Depression at 12 months	0.725*	0.201***	1.749	0.146^a^	Complete	7.58%
GSRS at 1 month						
Hunger pains severity	0.096*	0.977***	1.603	0.094^a^	Complete	5.54%
Acid regurgitation severity	0.147*	0.614***	1.703	0.090^a^	Complete	5.02%
GSRS at 6 months						
Hunger pains severity	0.128*	0.583**	1.820	0.074^a^	Complete	3.91%

*Note:* *p < 0.05; **p < 0.01; ***p < 0.001. The results are expressed in a log‐odds metric. All analyses were conducted using the PROCESS macro for SPSS (version 4.2; Andrew F. Hayes 2022), with bootstrapping techniques with 95% CI based on 5000 samples.

Abbreviations: GSRS, gastrointestinal symptom rating scale; IBS, irritable bowel syndrome; M, mediating variables.

^a^ Significant point estimates (*p* < 0.05) as determined by absence of zero within the confidence interval.

### 3.5 Mediating Variables: GI Symptoms at Baseline, 1 and 6 Months

The results of the mediation analysis revealed a significant indirect effect (a × b, as depicted in Figure 1A) of acid regurgitation at baseline (log‐odds metric = 0.090, 95% CI 0.001–0.182) and hunger pains at 1 month (log‐odds metric = 0.094, 95% CI 0.023–0.173), and hunger pains at 6 months (log‐odds metric = 0.074, 95% CI 0.016–0.149). Furthermore, the direct effect of COVID‐19 on post‐COVID‐19 IBS was not significant in the presence of the following mediators: Acid regurgitation at baseline (log‐odds metric = 1.703, 95% CI −0.343–3.749), hunger pains at 1 month (log‐odds metric = 1.603, 95% CI −0.452–3.658) and hunger pains also at 6 months (log‐odds metric = 1.819, 95% CI −0.218–3.858). Hence, acid regurgitation and hunger pains at 1 month and 6 months were the complete mediating variables between COVID‐19 and post‐COVID‐19 IBS (Tables 3 and S2).

## 4 Discussion

In this post hoc analysis, we attempted to explain the relationship between COVID‐19 and post‐COVID‐19 IBS. We hypothesized that the effect of COVID‐19 upon post‐COVID‐19 IBS is operated, completely or partially, through several clinical and psychological mediating variables. In our previous study on the relationship between COVID‐19 and postinfectious IBS (PI‐IBS) we reported three predictive factors of COVID‐19 IBS, namely, history of allergies (OR, 10.024; 95% CI, 1.766–56.891; *p* = 0.009), chronic intake of proton pump inhibitors (OR, 4.816; 95% CI, 1.447–16.025; *p* = 0.010), and dyspnea (OR, 4.157; 95% CI, 1.336–12.934; *p* = 0.014), which were significant after multivariate analysis [6]. Other studies assessing the rate of post‐COVID‐19 IBS [16, 17, 18, 19], instead, did not find any predictive factor except for the presence of GI symptoms at baseline.

In this mediation analysis, no laboratory test was found to be a mediating variable between the occurrence of COVID‐19 and the development of post‐COVID‐19 IBS. In a previous analysis, the role of inflammatory markers was not univocal [17]. In fact, in a recent study by Siyal et al., post‐COVID‐19 IBS was more likely in patients with higher levels of CRP and procalcitonin (PCT) during hospitalization, although only PCT remained significant after regression [17]. Our analysis, however, showed that while the levels of lymphocytes, platelets, creatinine, and IL‐6 were significantly lower, CRP levels were significantly higher in COVID‐19 patients compared to controls. These opposing trends likely reflect the nature of our control group, which consisted of hospitalized non‐COVID patients with varying comorbidities, some of whom had other underlying inflammatory conditions contributing to inflammatory variability. This heterogeneity may have masked the role of systemic inflammation as a possible mediator. Similarly, the widespread use of antibiotics in hospitalized patients, irrespective of COVID‐19 status, may have further obscured potential mediating effects on post‐COVID IBS, as antibiotic exposure was common in both groups. Alternatively, this suggests that systemic inflammation alone may not be the primary driver of post‐COVID IBS development and that other factors, such as localized gut inflammation, autonomic dysfunction, and physiological stress responses, may play a more substantial role.

Our analysis highlighted the importance of the mediation of psychological factors in the development of IBS. In fact, we have demonstrated how depression at 6 and 12 months completely mediates the relationship between COVID‐19 and post‐COVID‐19 IBS.

The causal relationship between COVID‐19 and depression has been extensively proven. The COVID‐19 Mental Disorders Collaborators concluded that the pandemic led to a 27.6% increase in cases of major depressive disorders [20, 21]. Another population‐based prospective study that examined individuals with anxiety and/or depression demonstrated that persons who had higher levels of anxiety and depression at the beginning of the study were considerably more prone to developing IBS [22, 23].

Therefore, we can speculate that in our cohort, COVID‐19 led to the development of depression, which, in turn, contributed independently to the development of IBS.

The predictive role of COVID‐19–related GI symptoms in the development of post‐COVID‐19 IBS has been demonstrated by various authors [16, 17, 18, 19, 24, 25, 26, 27, 28]. Our previous data showed that GI symptoms occurred more frequently in patients with COVID‐19 than in the control group (59.7% vs. 43.2%) [10]. It is not clear, however, which symptoms, when present, contribute to the development of COVID‐19 IBS. Our analysis showed that at baseline, no GI symptoms influenced the development of post‐COVID‐19 IBS. However, their persistence during follow‐up visits at 1 and 6 months contributes to the development of this DGBI. Specifically, at 1 month and 6 months, the presence of higher levels of hunger pain was able to explain, respectively, 5.54% and 3.91% of the total effect. Hunger pains may contribute to IBS development through multiple mechanisms. One possible explanation is that disruptions in gastric motility following COVID‐19 [29] may lead to altered gastric emptying and dysregulated hunger signaling, which could heighten visceral hypersensitivity [30]. Additionally, prolonged periods of fasting or disrupted eating patterns due to illness could exacerbate gut–brain axis dysfunction, further sensitizing the enteric nervous system [30, 31, 32]. Hunger pains may also serve as a marker of altered ghrelin secretion [33], and have been found to be significantly elevated in COVID‐19 patients even months after infection, potentially influencing appetite regulation, immune response, and gut motility [34].

Another symptom mediating the effect of COVID‐19 on post‐COVID‐19 IBS was acid regurgitation at 1 month. Acid regurgitation may serve as a mediator due to the interplay between SARS‐CoV‐2's impact on the GI system and the development of disorders DGBI. SARS‐CoV‐2 infects the GI tract by binding to ACE2 receptors, which are abundant in the esophageal and gastric mucosa. This can disrupt the normal function of the lower esophageal sphincter, leading to acid regurgitation. Additionally, the systemic inflammatory response and cytokine storm triggered by COVID‐19 may exacerbate esophageal sensitivity and impair gastric motility, further contributing to acid‐related symptoms. Acid regurgitation may, in turn, amplify gut–brain axis dysfunction by heightening visceral hypersensitivity and triggering central sensitization, processes central to IBS pathophysiology. This symptom may also reflect broader disruptions in the GI microbiome and motility caused by COVID‐19, which have been implicated in the development of IBS‐like symptoms. However, while PPIs effectively reduce acid exposure, they may also alter the gut microbiome, potentially promoting bacterial overgrowth and dysbiosis, which are known IBS risk factors. Given these effects, our findings do not support a universal recommendation for aggressive reflux treatment with PPIs as a preventive strategy for IBS.

Microbial dysbiosis can, in fact, participate in the COVID‐19 systemic inflammatory response and cytokine storm, leading to a more severe acute disease which can in turn cause more GI symptoms [8]. The relevance of the severity of the initial infection is confirmed by our finding of dyspnea as a mediator of post‐COVID‐19 IBS. The presence of dyspnea during the initial infection is a marker of a more severe disease and, as described in a large meta‐analysis by Klem et al. on PI‐IBS, more severe infections yield a higher risk of disease development after the initial insult [35].

The main strength of the study lies in its prospective nature and, therefore, its ability to make causal inference. Mediation analysis is based on the assumption of temporal precedence of the exposure, mediator, and outcome, which means that changes in the exposure to COVID‐19 are assumed to precede changes in each one of the clinical and psychological mediators, and that changes in the mediators are assumed to precede changes in the outcome (post‐COVID‐19 IBS). This analysis sheds light on the mediating factors that lead to the development of IBS, going beyond the simple linearity between variable and outcome, highlighting a possible extra pathophysiological pathway that would otherwise be overlooked.

There are several limitations in our investigation. The external validity and generalizability of the findings may have been influenced by several factors. First, the inclusion of patients from 14 countries and 36 centers introduced variability in hospitalization criteria and healthcare resource availability during the early pandemic. Additionally, the study focused exclusively on hospitalized COVID‐19 patients, excluding those with milder disease managed as outpatients. This narrow focus restricts the generalizability of the findings to individuals with severe COVID‐19, making it difficult to extrapolate the results to less severe cases or broader populations. Furthermore, the exclusion of 260 patients who did not complete the 12‐month follow‐up further contributed to selection bias, as these excluded patients exhibited distinct characteristics, such as higher creatinine and C‐reactive protein (CRP) levels and lower ferritin levels (Table S4), potentially representing a less symptomatic subgroup. Moreover, the control group consisted of hospitalized patients admitted for reasons unrelated to GI disorders. This choice, while pragmatic, may have introduced heterogeneity into the study cohort due to significant differences in baseline characteristics and comorbidities between the control and COVID‐19 groups. These differences could have diminished the statistical strength of our findings. A more specific control group consisting of patients hospitalized for non‐COVID‐19 infections or GI complaints might have served as a better comparator, minimizing variability and enhancing the interpretability of the results. Future studies should consider such refined control groups to strengthen the validity of comparisons. Together, these factors highlight the need for caution when extrapolating our findings to less severe COVID‐19 cases or broader, less symptomatic populations. Another key limitation of our study is that IBS was assessed as a binary outcome (presence or absence) based on Rome IV criteria, without grading its severity. As a result, we were unable to evaluate whether the identified mediators influence the severity of post‐COVID‐19 IBS, rather than just its occurrence. Future studies should incorporate validated tools for IBS severity assessment to further elucidate this aspect. Other limitations include the follow‐up duration, which was restricted to 1 year. Evidence suggests that GI symptoms following COVID‐19 infection can persist for longer periods [36], highlighting the need for extended observation to fully understand the mediating factors underlying PI‐IBS. Additionally, the use of the GSRS to evaluate GI symptoms prior to baseline, while aiming to minimize selection bias, may have introduced recall bias. Finally, the study was a post hoc analysis of previously published data, which could not account for major factors such as SARS‐CoV‐2 genomic variants or the widespread impact of mass vaccination campaigns.

In conclusion, our data suggest that there are psychological and clinical factors that link COVID‐19 to post‐COVID‐19 IBS. The occurrence of GI symptoms during the initial stages of COVID‐19 and their continued presence should raise awareness regarding the possibility of a new diagnosis of IBS. These factors could be potential targets for therapies aimed at treating or preventing depression.

## Author Contributions

G.B., G.M., and K.H.: designed the study; K.H.: conducted statistical analysis; G.M., G.B., and K.H.: validated and interpreted data; G.B, G.M. and K.H.: drafted the article; all authors collected data for the study, critically revised and approved the final version of the article.

## Conflicts of Interest

The authors declare no conflicts of interest.

## Supporting information


Appendix S1.

## Data Availability

The data that support the findings of this study are available on request from the corresponding author. The data are not publicly available due to privacy or ethical restrictions.

## References

[1] COVID‐19 Cases , “WHO COVID‐19 Dashboard.” n.d. https://data.who.int/dashboards/covid19/cases?n=o.

[2] “Overview. COVID‐19 Rapid Guidelines: Managing the Long‐Term Effect of COVID‐19. Guidance, NICE.” n.d.

[3] H. E. Davis , L. McCorkell , J. M. Vogel , and E. J. Topol , “Long COVID: Major Findings, Mechanisms and Recommendations,” Nature Reviews Microbiology 21 (2023): 133–146. 36639608 10.1038/s41579-022-00846-2 PMC9839201

[4] I. Kirchberger , C. Meisinger , T. D. Warm , A. Hyhlik‐Dürr , J. Linseisen , and Y. Goßlau , “Post‐COVID‐19 Syndrome in Non‐Hospitalized Individuals: Healthcare Situation 2 Years After SARS‐CoV‐2 Infection,” Viruses 15, no. 6 (2023): 1326, 10.3390/v15061326. 37376625 PMC10303962

[5] K. S. Cheung , I. F. N. Hung , P. P. Y. Chan , et al., “Gastrointestinal Manifestations of SARS‐CoV‐2 Infection and Virus Load in Fecal Samples From a Hong Kong Cohort: Systematic Review and Meta‐Analysis,” Gastroenterology 159, no. 1 (2020): 81–95, 10.1053/j.gastro.2020.03.065. 32251668 PMC7194936

[6] G. Marasco , C. Cremon , M. R. Barbaro , et al., “Post COVID‐19 Irritable Bowel Syndrome,” Gut 72, no. 3 (2022): 484–492, 10.1136/gutjnl-2022-328483. 36591612

[7] G. Marasco , M. Maida , C. Cremon , M. R. Barbaro , V. Stanghellini , and G. Barbara , “Meta‐Analysis: Post‐COVID‐19 Functional Dyspepsia and Irritable Bowel Syndrome,” Alimentary Pharmacology & Therapeutics 58, no. 1 (2023): 6–15, 10.1111/apt.17513. 37038318

[8] G. Marasco , M. V. Lenti , C. Cremon , et al., “Implications of SARS‐CoV‐2 Infection for Neurogastroenterology,” Neurogastroenterology and Motility 33, no. 3 (2021): e14104, 10.1111/nmo.14104. 33591607 PMC7995160

[9] D. Paramythiotis , E. Karlafti , M. Didagelos , et al., “Post‐COVID‐19 and Irritable Bowel Syndrome: A Literature Review,” Medicina 2023 59, no. 11 (2023): 1961, 10.3390/MEDICINA59111961. PMC10673195 38004010

[10] G. Marasco , C. Cremon , M. R. Barbaro , et al., “Prevalence of Gastrointestinal Symptoms in Severe Acute Respiratory Syndrome Coronavirus 2 Infection: Results of the Prospective Controlled Multinational GI‐COVID‐19 Study,” American Journal of Gastroenterology 117, no. 1 (2022): 147–157, 10.14309/ajg.0000000000001541. 34751672 PMC10337314

[11] “Diagnostic Testing for SARS‐CoV‐2.” (2024), https://who.int/publications/i/item/diagnostic‐testing‐for‐sars‐cov‐2.

[12] B. E. Lacy , F. Mearin , L. Chang , et al., “Bowel Disorders,” Gastroenterology 150, no. 6 (2016): 1393–1407.e5, 10.1053/j.gastro.2016.02.031. 27144627

[13] J. Svedlund , I. Sjödin , and G. Dotevall , “GSRS‐A Clinical Rating Scale for Gastrointestinal Symptoms in Patients With Irritable Bowel Syndrome and Peptic Ulcer Disease,” Digestive Diseases and Sciences 33, no. 2 (1988): 129–134, 10.1007/BF01535722. 3123181

[14] A. S. Zigmond and R. P. Snaith , “The Hospital Anxiety and Depression Scale,” Acta Psychiatrica Scandinavica 67, no. 6 (1983): 361–370, 10.1111/j.1600-0447.1983.tb09716.x. 6880820

[15] R. M. Baron and D. A. Kenny , “The Moderator‐Mediator Variable Distinction in Social Psychological Research: Conceptual, Strategic, and Statistical Considerations,” Journal of Personality and Social Psychology 51, no. 6 (1986): 1173–1182, 10.1037//0022-3514.51.6.1173. 3806354

[16] A. Nazarewska , K. Lewandowski , M. Kaniewska , M. Rosołowski , W. Marlicz , and G. Rydzewska , “Irritable Bowel Syndrome Following COVID‐19: An Underestimated Consequence of SARS‐CoV‐2 Infection,” Polish Archives of Internal Medicine 132, no. 11 (2022): 16323, 10.20452/PAMW.16323. 35997145

[17] M. Siyal , Z. Abbas , J. Ashraf , M. Ali Qadeer , and A. Altaf , “Incidence and Predisposing Factors for De Novo Post‐COVID‐19 Irritable Bowel Syndrome,” European Journal of Gastroenterology & Hepatology 35, no. 1 (2023): 59–63, 10.1097/MEG.0000000000002475. 36468570

[18] D. Zhang , C. Chen , Y. Xie , et al., “Post‐Infection Functional Gastrointestinal Disorders Following Coronavirus Disease‐19: A Prospective Follow‐Up Cohort Study,” BMC Infectious Diseases 23, no. 1 (2023): 422, 10.1186/S12879-023-08401-X. 37344782 PMC10286442

[19] R. Golla , S. Vuyyuru , B. Kante , et al., “Long‐Term Gastrointestinal Sequelae Following COVID‐19: A Prospective Follow‐Up Cohort Study,” Clinical Gastroenterology and Hepatology 21, no. 3 (2023): 789–796.e1, 10.1016/j.cgh.2022.10.015. 36273799 PMC9584755

[20] M. Daly and E. Robinson , “Depression and Anxiety During COVID‐19,” Lancet 399, no. 10324 (2022): 518, 10.1016/S0140-6736(22)00187-8. PMC8813060 35123689

[21] D. F. Santomauro , A. M. Mantilla Herrera , J. Shadid , et al., “Global Prevalence and Burden of Depressive and Anxiety Disorders in 204 Countries and Territories in 2020 due to the COVID‐19 Pandemic,” Lancet 398, no. 10312 (2021): 1700–1712, 10.1016/S0140-6736(21)02143-7. 34634250 PMC8500697

[22] K. G. Margolis , J. F. Cryan , and E. A. Mayer , “The Microbiota‐Gut‐Brain Axis: From Motility to Mood,” Gastroenterology 160, no. 5 (2021): 1486–1501, 10.1053/J.GASTRO.2020.10.066. 33493503 PMC8634751

[23] N. A. Koloski , M. Jones , and N. J. Talley , “Evidence That Independent Gut‐To‐Brain and Brain‐To‐Gut Pathways Operate in the Irritable Bowel Syndrome and Functional Dyspepsia: A 1‐Year Population‐Based Prospective Study,” Alimentary Pharmacology & Therapeutics 44, no. 6 (2016): 592–600, 10.1111/APT.13738. 27444264

[24] D. Noviello , A. Costantino , A. Muscatello , et al., “Functional Gastrointestinal and Somatoform Symptoms Five Months After SARS‐CoV‐2 Infection: A Controlled Cohort Study,” Neurogastroenterology and Motility 34, no. 2 (2022): e14187, 10.1111/NMO.14187. 34060710 PMC8209890

[25] R. Ebrahim Nakhli , A. Shanker , I. Sarosiek , et al., “Gastrointestinal Symptoms and the Severity of COVID‐19: Disorders of Gut–Brain Interaction Are an Outcome,” Neurogastroenterology and Motility 34, no. 9 (2022): e14368, 10.1111/NMO.14368. 35383423 PMC9115309

[26] E. Austhof , M. L. Bell , M. S. Riddle , et al., “Persisting Gastrointestinal Symptoms and Post‐Infectious Irritable Bowel Syndrome Following SARS‐CoV‐2 Infection: Results From the Arizona CoVHORT,” Epidemiology and Infection 150 (2022): e136, 10.1017/S0950268822001200. 35801302 PMC9343359

[27] F. Farsi , S. R. Zonooz , Z. Ebrahimi , et al., “The Incidence of Post‐Infectious Irritable Bowel Syndrome, Anxiety, and Depression in Iranian Patients With Coronavirus Disease 2019 Pandemic: A Cross‐Sectional Study,” Turkish Journal of Gastroenterology 33, no. 12 (2022): 1033–1042, 10.5152/TJG.2022.21651. PMC9797755 36098366

[28] Y. N. Wang , L. Y. Zhou , Y. H. Huang , M. Jiang , and C. Dai , “The Incidence and Predisposing Factors for Irritable Bowel Syndrome Following COVID‐19: A Systematic Review and Meta‐Analysis,” European Journal of Gastroenterology & Hepatology 36, no. 2 (2024): 168–176, 10.1097/MEG.0000000000002688. 38047738

[29] U. C. Ghoshal and U. Ghoshal , “Gastrointestinal Involvement in Post‐Acute Coronavirus Disease (COVID)‐19 Syndrome,” Current Opinion in Infectious Diseases 36, no. 5 (2023): 366–370, 10.1097/QCO.0000000000000959. 37606895

[30] K. Y. Huang , F. Y. Wang , M. Lv , X. X. Ma , X. D. Tang , and L. Lv , “Irritable Bowel Syndrome: Epidemiology, Overlap Disorders, Pathophysiology and Treatment,” World Journal of Gastroenterology 29, no. 26 (2023): 4120–4135, 10.3748/wjg.v29.i26.4120. 37475846 PMC10354571

[31] L. C. H. Yu , “Gastrointestinal Pathophysiology in Long COVID: Exploring Roles of Microbiota Dysbiosis and Serotonin Dysregulation in Post‐Infectious Bowel Symptoms,” Life Sciences 358 (2024): 123153, 10.1016/j.lfs.2024.123153. 39454992

[32] I. Łoniewski , K. Skonieczna‐Żydecka , J. Sołek‐Pastuszka , and W. Marlicz , “Probiotics in the Management of Mental and Gastrointestinal Post‐COVID Symptomes,” Journal of Clinical Medicine 11, no. 17 (2022): 5155, 10.3390/jcm11175155. 36079082 PMC9457065

[33] T. Kalli , T. Koutouratsas , G. Karamanolis , and M. Gazouli , “Ghrelin Gene Polymorphisms in Irritable Bowel Syndrome,” Digestion 102, no. 3 (2021): 313–318, 10.1159/000506306. 32294653

[34] J. Kuliczkowska‐Płaksej , A. Jawiarczyk‐Przybyłowska , A. Zembska , et al., “Ghrelin and Leptin Concentrations in Patients After SARS‐CoV2 Infection,” Journal of Clinical Medicine 12, no. 10 (2023): 3551, 10.3390/jcm12103551. 37240656 PMC10218943

[35] F. Klem , A. Wadhwa , L. J. Prokop , et al., “Prevalence, Risk Factors, and Outcomes of Irritable Bowel Syndrome After Infectious Enteritis: A Systematic Review and Meta‐Analysis,” Gastroenterology 152, no. 5 (2017): 1042–1054.e1, 10.1053/j.gastro.2016.12.039. 28069350 PMC5367939

[36] B. J. Elmunzer , O. S. Palsson , N. Forbes , et al., “Prolonged Gastrointestinal Manifestations After Recovery From COVID‐19,” Clinical Gastroenterology and Hepatology 22, no. 5 (2024): 1098–1107.e3, 10.1016/j.cgh.2023.11.009. 37995983

