# Supplementary material for: Psychological and Clinical Factors Mediate Post‐COVID‐19 Irritable Bowel Syndrome
Source: Neurogastroenterol Motil. 2025 May 15;38:e70079. doi: 10.1111/nmo.70079 (PMC13121868; doi:10.1111/nmo.70079)
Supplement: Supplementary file 1 — Appendix S1. [file NMO-38-e70079-s001.docx]

**Supplementary Table 1**. Comorbidities and chronic medication treatments of the study population.

|  | **Total population (n=623), n (%) or median (IQR)** | **Controls (n=188), n (%) or median (IQR)** | **COVID-19 patients (n=435), n (%) or median (IQR)** | **P-value** |
| --- | --- | --- | --- | --- |
| **Comorbidities** | | | | |
| Neurologic | 34 (5.5) | 19 (10.1) | 15 (3.4) | 0.002 |
| Cardiovascular | 184 (29.5) | 70 (37.2) | 114 (26.2) | 0.007 |
| Respiratory | 50 (8.0) | 21 (11.2) | 29 (6.7) | 0.076 |
| Liver | 30 (4.8) | 13 (6.9) | 17 (3.9) | 0.152 |
| Kidney | 32 (5.1) | 15 (8.0) | 17 (3.9) | 0.047 |
| Diabetes | 111 (17.8) | 46 (24.5) | 65 (14.9) | 0.006 |
| Metabolic other than  diabetes | 67 (10.8) | 27 (14.4) | 40 (9.2) | 0.067 |
| Musculoskeletal | 15 (2.4) | 7 (3.7) | 8 (1.8) | 0.164 |
| Psychiatric | 13 (2.1) | 7 (3.7) | 6 (1.4) | 0.071 |
| Gynecological | 3 (0.5) | 2 (1.1) | 1 (0.2) | 0.218 |
| Urological | 25 (4.0) | 12 (6.4) | 13 (3.0) | 0.072 |
| Rheumatological | 14 (2.2) | 5 (2.7) | 9 (2.1) | 0.769 |
| Allergies | 25 (4.0) | 12 (6.4) | 13 (3.0) | 0.072 |
| Autoimmune | 23 (3.7) | 11 (5.9) | 12 (2.8) | 0.067 |
| Neoplastic | 16 (2.6) | 9 (4.8) | 7 (1.6) | 0.028 |
| Psychological | 11 (1.8) | 4 (2.1) | 7 (1.6) | 0.742 |
| Hematological | 12 (1.9) | 6 (3.2) | 6 (1.4) | 0.199 |
| **Chronic medication intake with possible GI effect** | | | | |
| Proton pump inhibitor | 103 (16.5) | 47 (25.0) | 56 (12.9) | <0.001 |
| Non-steroidal  anti-inflammatory drugs | 41 (6.6) | 22 (11.7) | 19 (4.4) | 0.001 |
| Steroids | 20 (3.2) | 12 (6.4) | 8 (1.8) | 0.005 |
| Metformin | 36 (5.8) | 13 (6.9) | 23 (5.3) | 0.456 |
| Serotonin selective  reuptake inhibitors | 17 (2.7) | 7 (3.7) | 10 (2.3) | 0.421 |
| Antipsychotic | 6 (1.0) | 3 (1.6) | 3 (0.7) | 0.373 |
| Iron | 9 (1.4) | 5 (2.7) | 4 (0.9) | 0.138 |
| Fibrates | 4 (0.6) | 0 (0.0) | 4 (0.9) | 0.321 |
| ACE-I | 64 (10.3) | 25 (13.3) | 39 (9.0) | 0.114 |
| Beta-blockers | 83 (13.3) | 32 (17.0) | 51 (11.7) | 0.094 |
| Angiotensin-2 antagonist | 49 (7.9) | 12 (6.4) | 37 (8.5) | 0.420 |
| Lithium | 0 (0.0) | 0 (0.0) | 0 (0.0) | NA |
| Carbamazepine | 3 (0.5) | 2 (1.1) | 1 (0.2) | 0.218 |
| Furosemide | 25 (4.0) | 19 (10.1) | 6 (1.4) | <0.001 |
| 5-ASA | 7 (1.1) | 1 (0.5) | 6 (1.4) | 0.681 |
| Rifaximin | 2 (0.3) | 2 (1.1) | 0 (0.0) | 0.091 |
| Opiates | 4 (0.6) | 2 (1.1) | 2 (0.5) | 0.588 |
| Anticholinergics | 1 (0.2) | 1 (0.5) | 0 (0.0) | 0.302 |
| Verapamil | 4 (0.6) | 2 (1.1) | 2 (0.5) | 0.588 |
| Levothyroxine | 24 (3.9) | 11 (5.9) | 13 (3.0) | 0.111 |
| Cholestyramine | 1 (0.2) | 1 (0.5) | 0 (0.0) | 0.302 |
| Monoclonal antibodies | 1 (0.2) | 1 (0.5) | 0 (0.0) | 0.302 |
| Digoxin | 1 (0.2) | 0 (0.0) | 1 (0.2) | >0.999 |
| Dopaminergic agents | 2 (0.3) | 1 (0.5) | 1 (0.2) | 0.513 |
| H2 blockers | 4 (0.6) | 3 (1.6) | 1 (0.2) | 0.084 |
| Benzodiazepines | 21 (3.4) | 10 (5.3) | 11 (2.5) | 0.091 |
| Tricyclic antidepressant | 3 (0.5) | 1 (0.5) | 2 (0.5) | >0.999 |
| Antibiotics in the last  3 months | 182 (29.2) | 72 (38.3) | 110 (25.3) | 0.001 |
| Probiotics in the last  3 months | 65 (10.4) | 26 (13.8) | 39 (9.0) | 0.086 |

Controls were defined as patients without COVID-19 infection.

Abbreviations: ACE-I, ACE inhibitor; ALT, alanine amino transferase; 5-ASA, acid 5 amino-salicylic; AST, aspartate amino transferase; BMI, body mass index; GI, gastrointestinal; GERD, gastroesophageal reflux disease; GGT, gamma glutamil transferase; H. pylori, helicobacter pylori; IL-6, interleukin 6; INR, international normalized ratio; IQR, interquartile range; n, number; NA, not applicable

**Supplementary Table 2**. Simple mediation analyses for the relationship between COVID-19 and post COVID-19 IBS (for parametric mediating variables).

| Mediating variables (M) | Effect of COVID-19 on M (a) | Effect of M on post COVID-19 IBS (b) | Direct effect  (c`) | Indirect effect  (a x b) | Mediation effect | % of mediation effect of the total effect |
| --- | --- | --- | --- | --- | --- | --- |
| **Laboratory tests at baseline** | | | | | |  |
| Lymphocyte count (10^3^/mm^3^) | 0.925 | 0.018 | 1.938 | 0.016 | No |  |
| Platelets count (10^3^/mm^3^) | -42.8^***^ | 0.002 | 2.082^***^ | 0.068 | No |  |
| INR | -0.103^**^ | -0.001 | 1.761 | 0.0001 | No |  |
| Creatinine (mg/dl) | -0.252 | -0.576 | 1.933 | 0.145 | No |  |
| AST (UI/L) | -10.103 | 0.001 | 1.830 | -0.012 | No |  |
| ALT (UI/L) | -4.529 | -0.006 | 1.893 | 0.027 | No |  |
| GGT (UI/L) | -13.296 | -0.026 | 1.398 | 0.345 | No |  |
| Ferritin (mg/l) | -12.641 | -0.0003 | 13.861^*^ | 0.003 | No |  |
| IL-6 (pg/ml) | -302.903^***^ | -0.020 | 12.785 | 5.959 | No |  |
| C-reactive protein (mg/dl) | 2.655 | -0.002 | 1.826 | -0.005 | No |  |
| **Psychological disorders** | | | | | |  |
| Depression at 6 months | 0.784^*^ | 0.135^**^ | 1.817 | 0.106^†^ | Complete | 5.51% |
| Anxiety at 6 months | 0.310 | 0.128^*^ | 2.014 | 0.039 | No |  |
| Depression at 12 months | 0.725^*^ | 0.201^***^ | 1.749 | 0.146^†^ | Complete | 7.58% |
| Anxiety at 12 months | 0.529 | 0.159^**^ | 1.826 | 0.084 | No |  |
| **Gastrointestinal Symptom Rating Scale (GSRS) at baseline** | | | | | |  |
| Hunger pains severity | 0.240^*^ | 0.345^*^ | 1.803 | 0.083 | No |  |
| Nausea severity | 0.532^***^ | 0.069 | 1.930 | 0.037 | No |  |
| Heartburn severity | 0.146 | -0.227 | 1.988 | -0.033 | No |  |
| Acid regurgitation severity | 0.139^*^ | 0.131 | 1.944 | 0.018 | No |  |
| Borborygmus severity | 0.219^*^ | 0.049 | 1.951 | 0.011 | No |  |
| Abdominal distension severity | 0.110 | 0.333^*^ | 1.894 | 0.037 | No |  |
| Eructation severity | 0.075 | -0.080 | 1.960 | -0.006 | No |  |
| Increased flatus severity | 0.136 | 0.317^*^ | 1.870 | 0.043 | No |  |
| **Gastrointestinal Symptom Rating Scale (GSRS) at 1 month** | | | | | |  |
| Hunger pains severity | 0.096^*^ | 0.977^***^ | 1.603 | 0.094^†^ | Complete | 5.54% |
| Nausea severity | 0.112^*^ | 0.237 | 1.858 | 0.027 | No |  |
| Heartburn severity | 0.127^*^ | 0.557^**^ | 1.789 | 0.071 | No |  |
| Acid regurgitation severity | 0.147^*^ | 0.614^***^ | 1.703 | 0.090^†^ | Complete | 5.02% |
| Borborygmus severity | -0.001 | 0.533^**^ | 1.919 | -0.001 | No |  |
| Abdominal distension severity | 0.043 | 0.545^**^ | 1.922 | 0.024 | No |  |
| Eructation severity | -0.034 | 0.446 | 1.917 | -0.015 | No |  |
| Increased flatus severity | 0.139 | 0.188 | 1.863 | 0.026 | No |  |
| **Gastrointestinal Symptom Rating Scale (GSRS) at 6 months** | | | | | |  |
| Hunger pains severity | 0.128^*^ | 0.583^**^ | 1.820 | 0.074^†^ | Complete | 3.91% |
| Nausea severity | -0.007 | 0.603^***^ | 2.077^*^ | -0.004 | No |  |
| Heartburn severity | 0.006 | 0.700^***^ | 2.183^*^ | 0.004 | No |  |
| Acid regurgitation severity | 0.084 | 0.593^***^ | 1.933 | 0.050 | No |  |
| Borborygmus severity | 0.096 | 0.519^***^ | 1.925 | 0.050 | No |  |
| Abdominal distension severity | 0.047 | 0.699^***^ | 2.061 | 0.033 | No |  |
| Eructation severity | -0.005 | 0.304 | 1.974 | -0.001 | No |  |
| Increased flatus severity | 0.022 | 0.399^***^ | 1.953 | 0.009 | No |  |

^†^Significant point estimates (p<0.05) as determined by absence of zero within the confidence interval

^*^p<0.05; ^**^ p<0.01; ^***^ p<0.001.

The results are expressed in a log-odds metric.

All analyses were conducted using the PROCESS macro for SPSS (version 4.2; Andrew F. Hayes 2022), with bootstrapping techniques with 95% CI based on 5000 samples.

Abbreviations: ALT, alanine amino transferase; AST, aspartate amino transferase; GGT, gamma glutamyl transferase; IL-6, interleukin 6; INR, international normalized ratio; M, mediating variables

**Supplementary Table 3**. Simple mediation analyses for the relationship between COVID-19 and post COVID-19 IBS (for non-parametric mediating variables).

| **Mediating variables (M)** | 1^st^ step: OR of COVID-19 on M (a) | 2^nd^ step: OR of M on post COVID-19 IBS (b) | 3^rd^ step: OR of COVID-19 on post COVID-19 IBS (c`) | 4^th^ step: ORs of the adjusted model (b, c`) | **Interpretation** |
| --- | --- | --- | --- | --- | --- |
| **COVID-19's symptoms at baseline** | | | | | |
| Fever | 20.372^***^ | 7.021^*^ | 7.141^*^ | 4.638, 2.783 | No mediation effect |
| Fatigue | 10.338^***^ | 0.977 | 7.141^*^ | 0.610, 8.741^*^ | No mediation effect |
| Cough | 13.354^***^ | 3.536^*^ | 7.141^*^ | 2.344, 4.584 | No mediation effect |
| Myalgia | 9.087^***^ | 1.495 | 7.141^*^ | 1.047, 7.041 | No mediation effect |
| Dyspnea | 3.617^***^ | 4.461^**^ | 7.141^*^ | 3.561^*^, 5.172 | Complete mediation effect |
| Runny nose | 7.634^**^ | 1.051 | 7.141^*^ | 0.799, 7.237 | No mediation effect |
| Headache | 11.021^***^ | 0.216 | 7.141^*^ | 0.149, 9.368^*^ | No mediation effect |
| Anosmia | 33.422^***^ | 1.838 | 7.141^*^ | 1.272, 6.685 | No mediation effect |
| Dysgeusia | 18.408^***^ | 1.084 | 7.141^*^ | 0.762, 7.528 | No mediation effect |

^*^ p<0.05; ^**^ p<0.01; ^***^ p<0.001.

All analyses conducted using the Baron and Kenny's method with bootstrapping techniques with 95% CI based on 5000 samples.

M, mediating variables

**Supplementary Table 4.** Demographics, anamnestic characteristics, and psychological disorders of patients included in the study and those excluded due to lack of 12-month follow-up.

|  | **Included patients (n=623), n (%) or median (IQR)** | **Excluded patients (n=260), n (%) or median (IQR)** | **P value** |
| --- | --- | --- | --- |
| Age (years) | 51.0 (36.0-62.0) | 53.0 (36.3-67.0) | 0.059 |
| Sex, male | 369 (59.2) | 158 (61.2) | 0.762 |
| BMI (kg/m^2^) | 26.8 (24.0-30.9) | 26.0 (23.9-29.6) | 0.099 |
| Smoker |  |  | 0.774 |
| - No | 396 (64.0) | 168 (66.4) |  |
| - Current | 95 (15.3) | 35 (13.8) |  |
| - Former | 128 (20.7) | 50 (19.8) |  |
| Alcohol consumption | 114 (18.5) | 40 (15.7) | 0.379 |
| Physical activity (at least 30 minutes 3 times/week) | 202 (33.9) | 53 (20.9) | <0.001 |
| Laboratory tests |  |  |  |
| - Lymphocyte count (10^3^/mm)^3^ | 1.4 (0.9-2.1) | 1.5 (1.0-2.1) | 0.232 |
| - Platelet count (10^3^/mm)^3^ | 216.0 (166.0-273.8) | 214.0 (170.0-271.0) | 0.864 |
| - INR | 1.0 (0.9-1.1) | 1.0 (0.9-1.1) | 0.264 |
| - Creatinine (mg/dl) | 0.8 (0.7-1.0) | 0.9 (0.8-1.1) | 0.005 |
| - AST (UI/L) | 27.0 (19.0-42.0) | 26.0 (19.0-44.0) | 0.794 |
| - ALT (UI/L) | 27.0 (17.0-43.0) | 25.5 (18.0-41.0) | 0.858 |
| - GGT (UI/L) | 36.0 (21.0-70.0) | 36.0 (22.0-73.0) | 0.620 |
| - Ferritin (mg/l) | 242.0 (98.0-514.0) | 159.2 (64.5-435.3) | 0.008 |
| - IL-6 (pg/ml) | 28.6 (11.9-71.7) | 15.2 (10.4-36.6) | 0.273 |
| - C-reactive protein (mg/dl) | 3.3 (0.8-9.9) | 5.8 (1.2-23.5) | <0.001 |
| COVID-19 related symptoms (at baseline) |  |  |  |
| - Fever | 328 (52.6) | 123 (47.3) | 0.161 |
| - Fatigue | 260 (41.7) | 98 (37.7) | 0.293 |
| - Cough | 257 (41.3) | 107 (41.2) | >0.999 |
| - Myalgia | 168 (27.0) | 71 (27.3) | 0.934 |
| - Dyspnea | 157 (25.2) | 56 (21.5) | 0.263 |
| - Runny nose | 35 (5.6) | 21 (8.1) | 0.175 |
| - Headaches | 137 (22.0) | 43 (16.5) | 0.068 |
| - Anosmia | 117 (18.8) | 33 (12.7) | 0.030 |
| - Dysgeusia | 103 (16.5) | 28 (10.8) | 0.029 |
| Psychological disorders at 6 of follow-up |  |  |  |
| - Depression score at 6 months | 2.0 (0.0-5.0) | 2.0 (0.0-4.0) | 0.833 |
| - Anxiety score at 6 months | 3.0 (0.0-5.0) | 2.0 (0.0-5.0) | 0.518 |

Excluded patients included 260 patients with incomplete 12-month data.

Anxiety and depression were evaluated by HADS (Hospital Anxiety and Depression Scale).

Abbreviations: ALT, alanine amino transferase; AST, aspartate amino transferase; BMI, body mass index; GGT, gamma glutamyl transferase; IL-6, interleukin 6; INR, international normalized ratio; IQR, interquartile range; n, number
